# Molecular cloning and characterization of the *MsHSP17.7* gene from *Medicago sativa* L.

**DOI:** 10.1007/s11033-016-4008-9

**Published:** 2016-05-19

**Authors:** Zhen-yi Li, Rui-cai Long, Tie-jun Zhang, Qing-chuan Yang, Jun-mei Kang

**Affiliations:** Institute of Animal Sciences, Chinese Academy of Agricultural Sciences, Beijing, 100193 People’s Republic of China

**Keywords:** Heat shock protein, Homology cloning, *Medicago sativa*, RT-qPCR, Salt stress

## Abstract

Heat shock proteins (HSPs) are ubiquitous protective proteins that play crucial roles in plant development and adaptation to stress, and the aim of this study is to characterize the *HSP* gene in alfalfa. Here we isolated a small heat shock protein gene (*MsHSP17.7*) from alfalfa by homology-based cloning. *MsHSP17.7* contains a 477-bp open reading frame and encodes a protein of 17.70-kDa. The amino acid sequence shares high identity with MtHSP (93.98 %), PsHSP17.1 (83.13 %), GmHSP17.9 (74.10 %) and SlHSP17.6 (79.25 %). Phylogenetic analysis revealed that MsHSP17.7 belongs to the group of cytosolic class II small heat shock proteins (sHSP), and likely localizes to the cytoplasm. Quantitative RT-PCR indicated that *MsHSP17.7* was induced by heat shock, high salinity, peroxide and drought stress. Prokaryotic expression indicated that the salt and peroxide tolerance of *Escherichia coli* was remarkably enhanced. Transgenic *Arabidopsis* plants overexpressing *MsHSP17.7* exhibited increased root length of transgenic *Arabidopsis* lines under salt stress compared to the wild-type line. The malondialdehyde (MDA) levels in the transgenic lines were significantly lower than in wild-type, although proline levels were similar between transgenic and wild-type lines. *MsHSP17.7* was induced by heat shock, high salinity, oxidative stress and drought stress. Overexpression analysis suggests that *MsHSP17.7* might play a key role in response to high salinity stress.

**Electronic supplementary material:**

The online version of this article (doi:10.1007/s11033-016-4008-9) contains supplementary material, which is available to authorized users.

## Introduction

Plant HSPs normally participate in responses to drought, heat shock, salinity, heavy metals and peroxide stress [1]. HSPs act as molecular chaperones that bind other proteins to maintain steady-state target protein and promote the recovery of denatured proteins, which allows homeostasis of the internal environment during plant development and stress adaptation [2]. Small heat shock proteins (sHSPs, 15–42 kDa) form 200–800 kDa multimeric chaperone complexes [3] and are divided into six classes based on sequence similarity and cellular location. Class CI, CII and CIII sHSPs localize to the cytosol or nucleus [4], as well as the endoplasmic reticulum, mitochondria, and membranes [5]. sHSPs just bind to misfolded or denatured protein substrates, whereas refolding steps are mediated by Hsp70/Hsp100 complexes [6], preventing irreversible unfolding or aberrant protein aggregation [7]. Their ability to prevent irreversible protein aggregation and to resolubilize aggregated proteins allows native proteins to remain in a stable state. Thus, sHSPs have been described as the ‘paramedics of the cell’ [8, 9].

Several studies have shown that numerous plant sHSPs participate in the development of nutritive tissue, embryogenesis, germination and fruit production [10]. In addition, sHSP plays a significant role in the response to abiotic stresses. The *ArabidopsisAtHsp15.7* gene is expressed at very low levels in a normal environment but is highly expressed upon heat shock or oxidative stress [11]. A previous study demonstrated that overexpression of *sHSP17.7* increased drought tolerance in transgenic rice seedlings [7], and Lee et al. found that overexpression of the *MsHSP23* gene enhanced salinity and arsenic tolerance in transgenic tobacco [3].

Alfalfa (*Medicago sativa* L.) is an important leguminous forage plant and is cultivated worldwide because of its high nutritional value and yield. Adverse external conditions, such as salinity, drought, high temperature and other types of stress, have a negative impact on the yield and quality of alfalfa. Therefore, improved stress resistance is a priority in breeding efforts to produce novel varieties of alfalfa that are better adapted to specific local environments and varying climate conditions. Here, we report the characterization and function of *MsHSP17.7* gene from alfalfa, a possible candidate gene for stress tolerance in *M. sativa*.

## Materials and methods

### Plant materials and growth conditions

*M. sativa* L. cv. Zhongmu No.1 and *Arabidopsis thaliana* (ecotype; Col-0) were used for gene cloning and genetic modifications. Thirty alfalfa seedlings per pot were cultured in a Hoagland hydroculture system in a plant growth chamber with 60 % humidity, a temperature of 24 °C, and a 16 h light/8 h dark cycle. Wild-type *A. thaliana* was cultured in pots containing a vermiculite/soil (1:3) mixture under the conditions described above.

### Cloning of the *MsHSP17.7* gene and bioinformatics analysis

Homology-based cloning was performed to obtain the open reading frame (ORF) of *MsHSP17.7*. Plant total RNA was extracted from alfalfa using the MiniBEST plant RNA extraction kit (Takara Biotech Co., Ltd., Dalian, China), and cDNA was subsequently obtained using the PrimeScript RT-PCR Kit (Takara). MsHSP-F (sequence: 5′-CCTCCCATAATCTTCCAACCAC-3′) was used as the sense primer, and MsHSP-R (sequence: 5′-CAAAAAACCATTGCCACACACG-3′) was used as the antisense primer. DNA fragment were cloned using alfalfa cDNA as the template by ordinary PCR. The obtained DNA fragment was then ligated into the pEASY-T1 vector, which was purified from positive *Escherichia coli* colonies containing the *MsHSP17.7* gene and the insert was sequenced.

We analyzed the sequence using the abc website (http://abc.cbi.pku.edu.cn/). The ORF was found by plotorf ([v 6.0.1]; Alan Bleasby, European Bioinformatics Institute, UK) and translated into an amino acid sequence. The following analyses were performed: protein hydrophobicity (Protscale, http://web.expasy.org/protscale/), signal prediction (SignalP 4.1, http://www.cbs.dtu.dk/services/SignalP/), transmembrane motif prediction (TMHMM, http://www.cbs.dtu.dk/services/TMHMM-2.0/), protein secondary structure analysis (Garnier [v6.0.1]; William Pearson, European Bioinformatics Institute, UK), subcellular location prediction (ProtComp, http://www.softberry.com) and multiple sequence alignment (DNAMAN 6.0; Lynnon Biosoft, USA). The phylogenetic tree of MsHSP17.7 was constructed using MEGA5.1 [12] software.

### Subcellular localization of the *MsHSP17.7*

The coding sequence of *MsHSP17.7* was amplified from plasmid pEASY-T1 using the forward primer pA7-F (5′-CCGCTCGAGATGGATTTCAGGCTAATGGGT-3′; the *Xho*I site is underlined) and reverse primer pA7-R (5′-CGGACTAGTAGCAACCTTAACCTCAATAGT-3′; the *Spe*I site is underlined). Then, the fragment was digested with *Xho*I and *Spe*I and ligated into the similarly digested vector pA7-GFP (Supplementary Fig. 4) which contains the CaMV 35S-promoter and the green fluorescent protein (GFP) gene. pA7-GFP and MsHSP17.7-GFP gene fusion plasmids were transformed into onion epidermal cells using a particle gun (PDS1000/He; Bio-Rad, USA), and the bombarded tissues were incubated on a fresh plate for 16 h in dark environment. Then the bombarded onion epidermal cells were placed in 200 mM NaCl for 5–10 min for plasmolysis. Cells were visualized with a confocal laser-scanning microscope (TE2000-E; Nikon, Japan).

### Expression analysis of *MsHSP17.7*

To investigate the expression pattern of *MsHSP17.7* in alfalfa under heat shock, high salinity, oxidative stress and drought stress, 25-day-old alfalfa seedlings were treated over a 24-h period to induce heat shock (37 °C), salt stress (200 mM NaCl), oxidative injury (15 mM H_2_O_2_) or drought conditions (200 g l^−1^ polyethylene glycol (PEG) 6000) in Hoagland solution. Three alfalfa seedlings were randomly selected at 0, 2, 4, 8, 12 and 24 h, and total RNA was extracted from roots, stems and leaves. Subsquently cDNA was reversely transcribed using the PrimeScript RT-PCR Kit (Takara) described above. Then quantitative reverse-transcription PCR (RT-qPCR) were performed on roots, stems and leaves of alfalfa. The specific primers *MsHSP17.7* qhsp-f (5′-CACCACATAATGGACCTCACAGAT-3′) and qhsp-r (5′-TGATGTCACCTGATTTCAACCCTG-3′) were used in assays. The alfalfa *β*-*actin* gene (GenBank: JQ028730.1) was used as an internal control with the primers qact-f (5′-CAAAAGATGGCAGATGCTGAGGAT-3′) and qact-r (5′-CATGACACCAGTATGACGAGGTCG-3′). Then quantitative reverse transcription PCR (RT-qPCR) reactions were performed on roots, stems and leaves. The RT-qPCR protocol was performed as specified by the manufacturer’s instructions for the SYBR Primix Ex Tap II kit (Takara). The mean threshold cycle (Ct) was used as a reference value to calculate the level of each mRNA. Three biological replicates and per replicate contains three alfalfa seedlings were performed. The variance was subjected to a least significant difference (LSD) test using SAS software (version 9.13).

### Expression of *MsHSP17.7* in *E. coli* and salinity and oxidative stress survival assays

The complete *MsHSP17.7* ORF was amplified with forward primer EHSP-F (5′-ATGGATTTCAGGCTAATGGGTTTGG-3′) and reverse primer EHSP-R (5′-TCAAGCAACCTTAACCTCAATAGTCT-3′) and ligated into a prokaryotic expression vector (pEASY-E2, Novagen, USA) to generate the expression plasmid pEASY-E2/MsHSP17.7. Then, the plasmid was transformed into the *E. coli* strain Transetta DE3, which was cultured at 37 °C with shaking at 200 rpm. During log phase, 1 mM IPTG was added to bacterium solution to induce protein expression. The bacterial suspension was harvested by centrifugation for 0, 3, 5, 7 and 9 h, and the fusion protein was separated by 12 % SDS-PAGE and Coomassie brilliant blue (CBB) staining.

Tolerance to salt and oxidative stresses were tested by growing bacteria in LB medium supplemented with 100 mM NaCl and 15 mM H_2_O_2_. Then, *E. coli* cells expressing the pEASY-E2/MsHSP17.7 plasmid were incubated at 37 °C with shaking at 180 rpm. Empty vector-transfected *E. coli* was used as the control, and the growth rate was determined by measuring the OD600 value at 0, 2, 4, 6, 8, 10, 12 and 24 h [3].

### Construction of the plant expression vector and generation of transgenic *Arabidopsis*

*MsHSP17.7* cDNA containing *Xba*I and *BamH*I restriction sites was cloned with the primers pBI-F (5′-TGCTCTAGAATGGATTTCAGGCTAATGGGT-3′, *Xba*I site is underline) and pBI-R (5′-CGGGATCCAGCAACCTTAACCTCAATAGTC-3′, *BamH*I site underline). The *Xba*I-*BamH*I fragment was inserted into pBI121 encoding the CaMV 35S promoter. Subsequently, the pBI121-35S-MsHSP17.7 recombinant vector was transformed into *Agrobacterium* GV3101 using the freeze–thaw method. Then, *Agrobacterium*-mediated transformation of *Arabidopsis* was performed with the floral dip method [13]. The seeds obtained were screened on 1/2 MS medium with 50 mg l^−1^ kanamycin. The transformed *Arabidopsis* seedlings were transplanted into pots under the conditions described above.

To identify transgenic *A. thaliana*, genomic PCR and RT-PCR analyses were performed comparing wild-type and transgenic lines. The primers pBI-F and pBI-R were used to amplify the *MsHSP17.7* gene using the genomic gene in *A. thaliana* plants as a template for PCR. Then, At-act-F (5′-GAAGTCTTGTTCCAGCCCTCGTTTG-3′) and At-act-R (5′-GAACCACCGATCCAGACACTGTACT-3′) were used to amplify the *A. thalianaactin 2* gene (GenBank: NM_112764.3) based the cDNA template as a control. Additionally, pBI-F and pBI-R were used to amplify *MsHSP17.7* from the cDNA template. In this experiment, T3 transgenic *Arabidopsis* homozygous lines, T31 and T37, were randomly selected and used throughout the study.

To identify the *MsHSP17.7* was integrated into the position of the genome in *A. thaliana*, thermal asymmetric interlaced PCR (TAIL-PCR) was performed in this assay. The primers and cycling conditions of experiment protocol was described as Liu et al.’s paper [14]. Specific primer TR1 (5′-TGCATGACGTTATTTATGAGATGGGTT-3′) or TL1 (5′-TAGGGTTCCTATAGGGTTTCGCTCA-3′) was used in primary reaction, specific primer TR2 (5′-TATGATTAGAGTCCCGCAATTATACA-3′) or TL2 (5′-GTGTTGAGCATATAAGAAACCCTTAG-3′) was used in secondary reaction, and TR3 (5′-CTAGGATAAATTATCGC-3′) or TL3 (5′-CCTAAAACCAAAATCCAG-3′) was used in tertiary reaction [14].

### Analysis of transgenic *Arabidopsis* under stress conditions

For salt stress treatment, 15 seeds were randomly taken from T3 transgenic lines and wild-type *A. thaliana*. *Arabidopsis* were germinated on 1/2 MS medium containing 150 mM NaCl for 12 days, and the lengths of their roots were measured. As a control, 4-week wild-type *Arabidopsis*, then NaCl was added to the Hoagland solution to obtain a final concentration of 200 mM NaCl. At 36 h, the MDA and proline contents were measured. The MDA content was determined using the thiobarbituric acid (TBA) reaction, as described by Heath and Packer (1968). Three technical replicates and per replicate contains three *Arabidopsis* were performed. The variance was subjected to a LSD test using SAS software (version 9.13).

## Results

### Cloning and molecular characterization of *MsHSP17.7*

A 686 bp cDNA fragment containing a 477 bp ORF was amplified from alfalfa by homology cloning and designated *MsHSP17.7* (GenBank accession: KJ621408). The gene encoded a 158 amino acid protein with a molecular weight of 17.67 kDa. The theoretical isoelectric point was 5.789, which is characteristic of an acidic protein. The result from the ProtScale analysis indicated that most of the amino acids were hydrophilic (Supplementary Fig. 2A); therefore, MsHSP17.7 was deemed a hydrophilic protein. SignalP-4.1 identified no signal peptides in MsHSP17.7 (Supplementary Fig. 2B). In addition, the MsHSP17.7 protein was predicted to encode no transmembrane structures by TMHMM (Supplementary Fig. 2C). The protein secondary structure was predicted by Garnier [v6.0.1] to consist of 56.3 % α-helix, 26.1 % β-fold, 10.6 % β-corner, and 18.3 % random coil. Online software (ProtComp, http://www.softberry.com) indicated that the MsHSP17.7 protein was likely to localize to the cytoplasm.

**Fig. 1:**
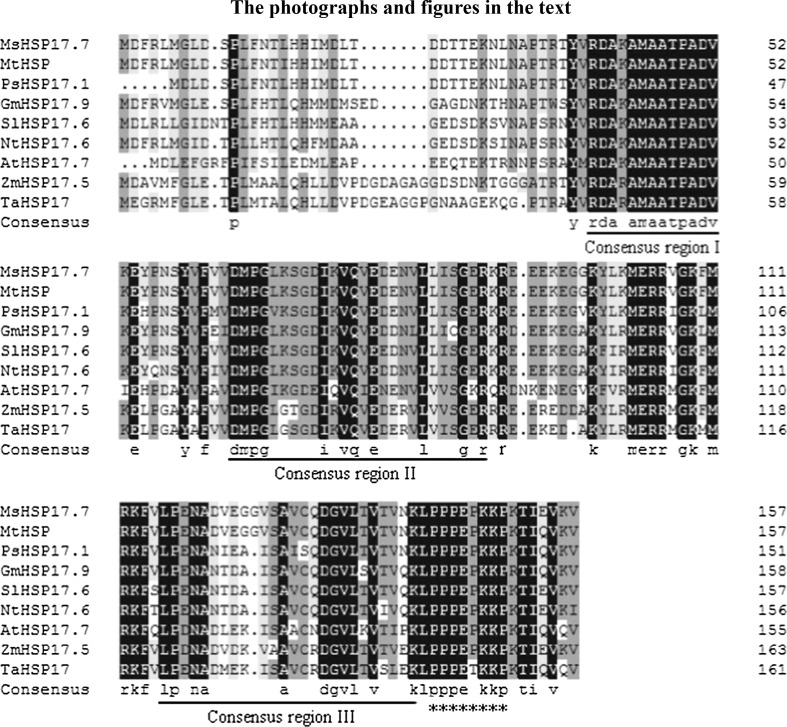
Multiple sequence alignment of MsHSP17.7 with other plant sHSPs. *Black* indicates that 100 % of the nine sequences have the same amino acid at a given position, *pink* indicates that 75 % of the sequences are conserved, and *blue* indicates that 50 % of the sequences are conserved. Conserved motifs are underlined. *Asterisk* indicates a polyproline motif. The accession numbers of the sHSPs and its similarity are as follows, MsHSP17.7 (*Medicago sativa*, A0A060CW40); MtHSP (*Medicago truncatula*, G7J8C7), 93.98 %; PsHSP17.1 (*Pisum sativum*, P19242), 83.13 %; GmHSP17.9 (*Glycine max*, P05477), 74.10 %; SlHSP17.6 (*Solanum lycopersicum*, Q96489), 79.25 %; Nthsp17.6 (*Nicotiana tabacum*, A0A077DBK4), 74.70 %; AtHAP17.7 (*Arabidopsis thaliana*, O81822), 56.63 %; ZmHSP17.5 (*Zea mays*, B6SJE9), 65.66 %; TaHSP17 (*Triticum aestivum*, A0A077RX64), 63.25 %. (Color figure online)

**Fig. 2:**
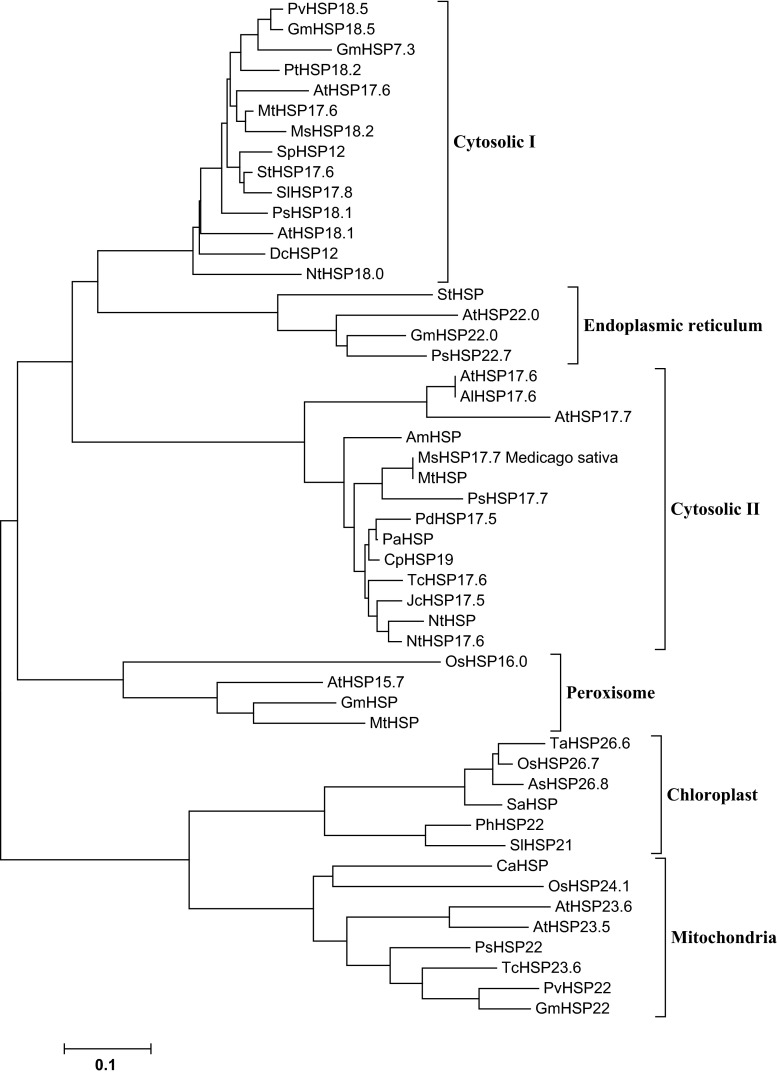
Neighbor-joining phylogenetic tree of MsHSP17.7 and sHSPs from other plant species. The phylogenetic tree was constructed based on similarities with 50 plant sHSPs, which divided the sHSP gene family into six clades. Amino acid sequences used in the analysis were retrieved from GenBank or EMBL. Their database accession numbers are as follows: AtHSP18.1 (*Arabidopsis thaliana*, P19037); NtHSP18.0 (*Nicotiana tabacum*, A0A068LKK5); PvHSP18.5 (*Phaseolus vulgaris*, T2DN13); DcHSP18.0 (*Daucus carota*, P27397); GmHSP18.5 (*Glycine max,* P05478); GmHSP17.3 (*Glycine max*, P02519); PtHSP18.2 (*Populus trichocarpa*, B9HHJ3); PsHSP18.1 (*Pisum sativum*, P19243); AtHSP17.6 (*Arabidopsis thaliana*, Q9ZW31); MtHSP17.6 (*Medicago truncatula*, Q2HTU2); MsHSP18.2 (*Medicago sativa*, P27880); StHSP17.6 (*Solanum tuberosum*, W5XNJ3); SlHSP17.8 (*Solanum lycopersicum*, P30221); SpHSP17.6 (*Solanum peruvianum*, O82012); StHSP (*Solanum tuberosum*, Q41218); AtHSP22.0 (*Arabidopsis thaliana*, Q38806); GmHSP22.0 (*Glycine max*, P30236); PsHSP 22.7 (*Pisum sativum*, P19244); PdHSP17.5 (*Prunus dulcis*, Q9XGS6); PlHSP (*Prunus salicina*, C9EIM5); CpHSP19 (*Citrus paradisi*, Q84LP5); TcHSP17.6 (*Theobroma cacao*, A0A061GJC5); JcHSP17.5 (*Jatropha curcas*, D5JG84); NtHSP (*Nicotiana tabacum*, Q53E18); NtHSP17.6 (*Arabidopsis thaliana*, A0A077DBK4); AmHSP (*Ammopiptanthus mongolicus*, S5TJ94); PsHSP17.1 (*Pisum sativum*, P19242); MtHSP (*Medicago truncatula*, G7J8C7); AlHSP17.7 (*Arabidopsis thaliana*, O81822); AtHSP17.6 (*Arabidopsis thaliana*, P29830); AtHSP17.6 (*Arabidopsis thaliana*, P29830); OsHSP16.0 (*Oryza sativa*, Q652V8); AtHSP15.7 (*Arabidopsis thaliana*, Q9FHQ3); GmHSP (*Glycine max*, B0M1A7); MtHSP (*Medicago truncatula*, G7KG40); TaHSP26.6 (*Triticum aestivum*, Q9SBB7); OsHSP26.7 (*Oryza sativa*, Q10P60); AsHSP26.8 (*Agrostis stolonifera*, Q8GV37); SaHSP (*Spartina alterniflora*, J7H8N1); PhHSP22 (*Petunia hybrida*, P30222); SlHSP21 (*Solanum lycopersicum*, Q95661); CaHSP (*Capsicum annuum*, D9IAX1); OsHSP24.1 (*Oryza sativa*, Q6Z7V2); AtHSP23.6 (*Arabidopsis thaliana*, Q96331); AtHSP23.5 (*Arabidopsis thaliana*, Q9FGM9); PsHSP22 M (*Pisum sativum*, P46254); TcHSP23.6 (*Theobroma cacao*, A0A061FZB7); PvHSP22 (*Phaseolus vulgaris*, V5N8V1); GmHSP22 (*Glycine max*, Q39818)

A multiple sequence alignment of the deduced MsHSP17.7 protein is shown in Fig. 1. MsHSP17.7 shares high protein sequence identity with MtHSP (93.98 %), PsHSP17.1 (83.13 %), GmHSP17.9 (74.10 %) and SlHSP17.6 (79.25 %), and it shares higher sequence similarity with dicotyledons compared with monocotyledons. Based on a comparison with cytosolic class II sHSP sequences, a unique domain (RDAKAMAATPADV) was found in the N terminus (Fig. 1). A conserved C-terminal domain (α-Crystallin domain, ACD) of approximately 90 amino acids contained consensus regions II and III. Additionally, a polyproline motif PPPEPKKP was identified at the C-terminus [15–17]. A phylogenetic tree showed that MsHSP17.7 was identified as a member of the plant cytosolic class II sHSPs (Fig. 2).

### Subcellular localization of the *MsHSP17.7*

**Fig. 3:**
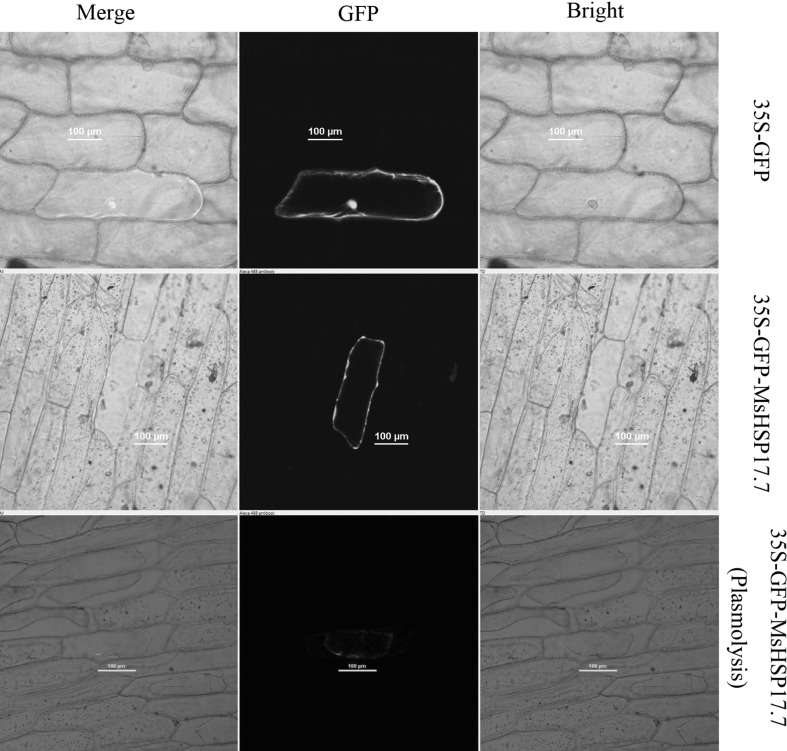
Subcellular localization of the MsHSP17.7-GFP fusion in onion epidermal cells. GFP fluorescence was distributed throughout the entire cell in cells expressing the GFP empty vector. GFP fluorescence was localized to the cytoplasm of cells expressing the MsHSP17.7-GFP fusion protein. Plasmolysis of cells indicated MsHSP17.7-GFP fluorescence accumulated mainly in the cytoplasm instead of the cell wall. Bar = 100 μm

GFP or the MsHSP17.7-GFP fusion protein was transiently expressed in onion epidermal cells. As shown in Fig. 3, the MsHSP17.7-GFP fusion protein accumulated mainly in the cytoplasm, whereas GFP alone was distributed throughout the entire cell. In addition to, the plasmolysis of onion cells indicated that MsHSP17.7-GFP fluorescence accumulated mainly in the cytoplasm instead of cell wall. This result was consistent with the prediction that *MsHSP17.7* by ProtComp online.

### Expression of *MsHSP17.7* in alfalfa

**Fig. 4:**
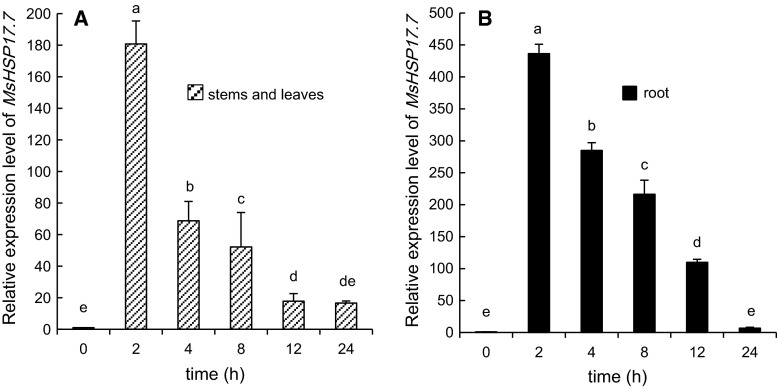
RT-qPCR analysis of *MsHSP17.7* in shoot (**A**) and root (**B**) tissues in response to heat shock (37 °C) treatments at different time intervals. After 0–24-h of heat treatment, the transcript abundance from 2-week-old alfalfa seedlings was determined. *Vertical* *bars* indicate the mean ± SE of three biological independent experiments. The *same* *letter* is used to indicate no significant difference according to the LSD *t* test (*P* < 0.05)

**Fig. 5:**
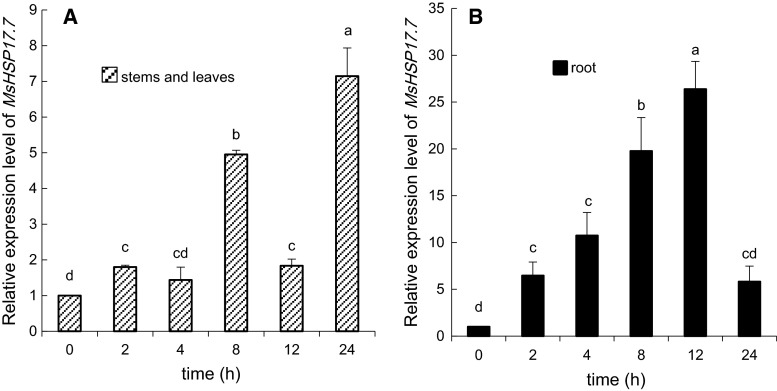
RT-qPCR analysis of *MsHSP17.7* in shoot (**A**) and root (**B**) tissues in response to 200 mM NaCl treatment at different time intervals. Transcript abundance from 2-week-old alfalfa seedlings was detected during a 24 h treatment. *Vertical bars* indicate the mean ± SE of three biological independent experiments. The *same* *letter* is used to indicate no significant difference according to the LSD *t* test (*P* < 0.05)

**Fig. 6:**
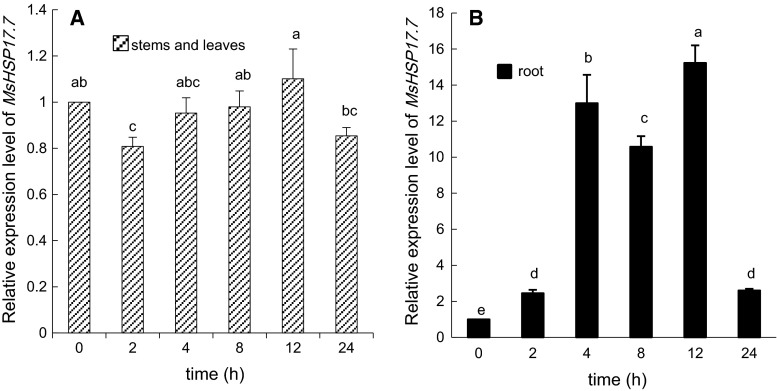
RT-qPCR analysis of *MsHSP17.7* in shoot (**A**) and root (**B**) tissues in response to 15 mM H_2_O_2_ oxidative treatments at different time intervals. Transcript abundance from 2-week-old alfalfa seedlings was detected during a 24 h treatment. *Vertical bars* indicate the mean ± SE of three biological independent experiments. The *same* *letter* is used to indicate no significant difference according to the LSD *t* test (*P* < 0.05)

**Fig. 7:**
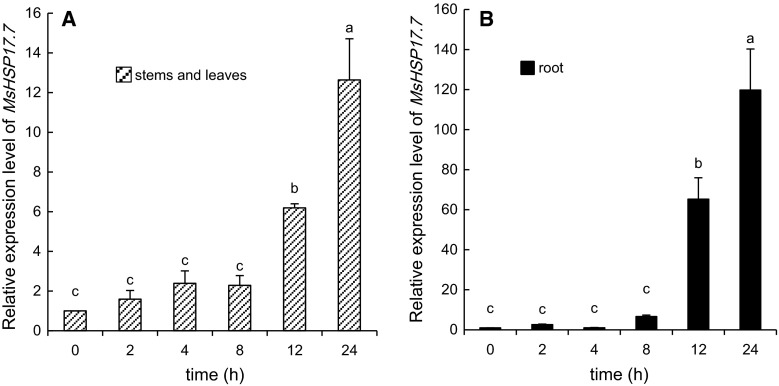
RT-qPCR analysis of *MsHSP17.7* in shoot (**A**) and root (**B**) tissues in response to osmotic (200 g l^−1^ PEG 6000) stress at different time intervals. Transcript abundance after a 0–24-h treatment of two-week-old alfalfa seedlings was detected. *Vertical bars* indicate the mean ± SE of three biological independent experiments. The *same* *letter* is used to indicate no significant difference according to the LSD *t* test (*P* < 0.05)

The relative expression levels of *MsHSP17.7* mRNA under different stress conditions are shown in Figs. 4, 5, 6 and 7. Under heat shock stress (Fig. 4), the pattern of expression in stems and leaves was consistent with that in roots. After a rapid increase after 2 h, the expression of mRNA *MsHSP17.7* decreased as treatment time increased. At 2 h, the level of *MsHSP17.7* mRNA in the above-ground parts was 180.7-fold higher than in the control, and the expression in the underground parts was 436.5-fold higher than in the control.

As shown in Fig. 5, the mRNA expression levels were higher under salt stress in the stems and leaves of stressed plants compared to those of control plants, except after 4 h of stress induction. However, there were no significant differences at 2, 4 and 12 h. The mRNA expression level was 4-fold higher in the above-ground tissues of stressed plants than in those of the control plants at 8 h. The expression level was the highest at 24 h (7-fold higher). The mRNA expression level of *MsHSP17.7* in the root tissue gradually increased with treatment time until 12 h, at which time the level was 25-times higher than in the roots of the control plants. The mRNA expression level in the root suddenly decreased at 24 h of treatment to a level that was 5-fold higher than that of the control, whereas there was no obvious difference between expression levels at 0 and 24 h (Fig. 5B).

Upon peroxide stress treatment (Fig. 6A), the expression levels of *MsHSP17.7* mRNA in the stems and leaves corresponded to those of the control at 4, 8 and 12 h. The expression levels at 2 and 24 h were slightly lower than those of the control at 0 h. The *MsHSP17.7* mRNA expression pattern in alfalfa root was completely different, increasing 1.5-, 12.0-, 9.5-, 14.2- and 1.6-fold at 2, 4, 8, 12, and 24 h (Fig. 6B).

The expression pattern of *MsHSP17.7* in stems and leaves was consistent with that in roots under osmotic stress (Fig. 7A, B). *MsHSP17.7* expression levels in the whole plant were not significantly different from those of the control from 0 to 8 h. After 8 h, the expression levels increased. At 24 h, the *MsHSP17.7* mRNA expression level peaked at a 12-fold increase over controls in stems and leaves and at a 119-fold increase over controls in roots.

### Expression of *MsHSP17.7* in *E. coli* and salinity and oxidative stress survival assays

**Fig. 8:**
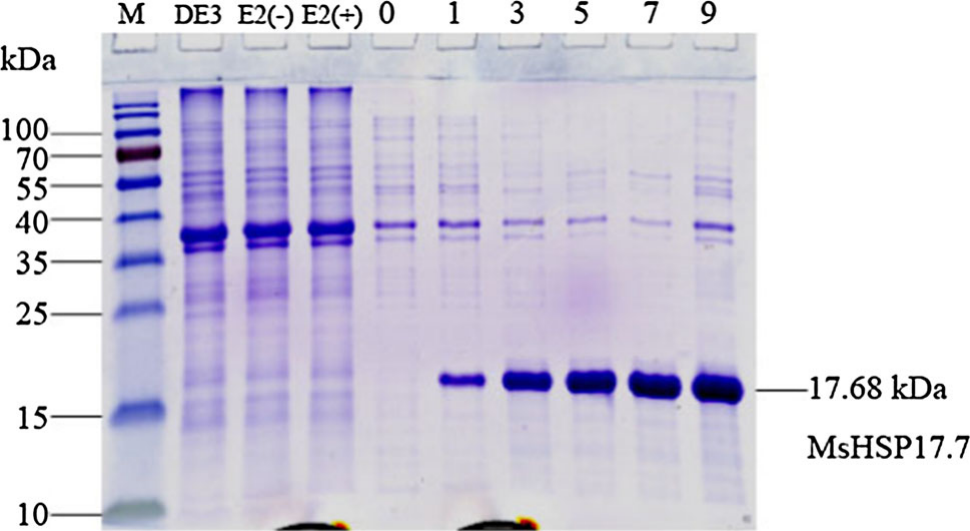
Coomassie-stained gel was used in SDS-PAGE analysis of MsHSP17.7 fusion protein expression in *E. coli*. *Lane M* protein ladder, *lane DE3* *E. coli* strain DE3 total protein, *lane E2* (−) pEASY-E2 total protein without ITPG, *lane E2* (+) pEASY-E2 total protein with ITPG induction, *lane 0* MsHSP17.7 soluble protein induction 0 h (+ITPG), *lane 1, 3, 5, 7, 9* MsHSP17.7 soluble protein induction 1, 3, 5, 7, 9 h (+ITPG)

Under the T7 promoter, the MsHSP17.7 protein was abundantly expressed in *E. coli* (Fig. 8), and SDS-PAGE analysis clearly showed a 17.70 kDa band. With the increased time of induction, the amount of IPTG-induced MsHSP17.7 expression was gradually increased (Fig. 8).

**Fig. 9:**
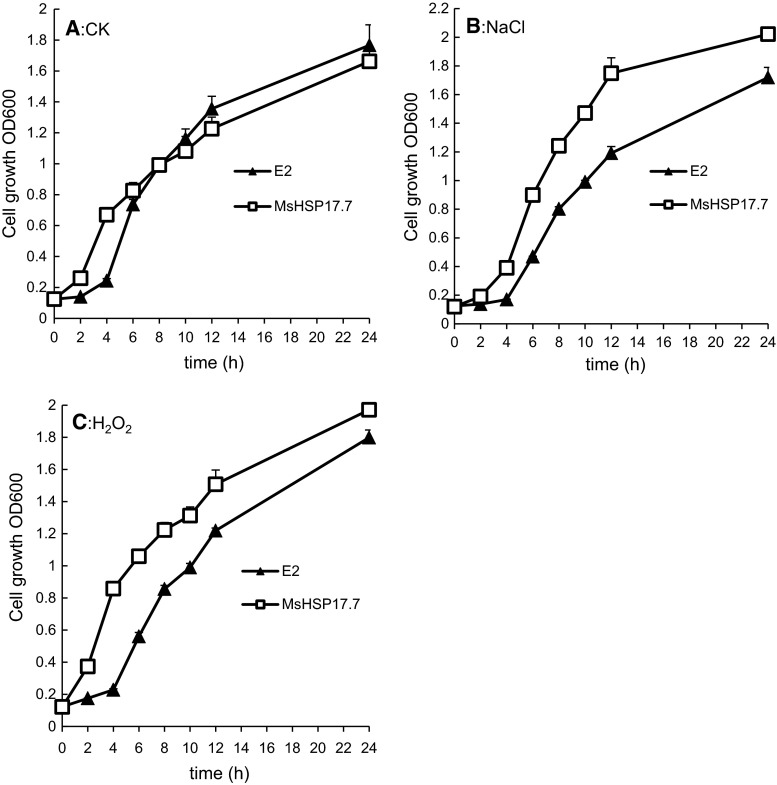
The effects of MsHSP17.7 expression on the growth of *E. coli* under salt and peroxide stress conditions. Compared to the control of *E. coli* in normal condition (**A**), *E. coli* cell growth after 100 mM NaCl treatment (**B**) and 15 mM H_2_O_2_ treatment (**C**) was determined. E2 denoted *E. coli* with pEASY-E2 and represented control in (**A–C**). MsHSP17.7 denoted *E. coli* with *MsHSP17.7*. The mean value of each transgenic line represented a statistically significant difference with respect to the control, as determined by the LSD *t* test (*P* < 0.05). *Vertical bars* indicate the mean ± SE of three biological independent experiments

To evaluate whether *MsHSP17.7* expression increased the capacity to resist salinity or peroxide stress in model microorganisms, the effects of *MsHSP17.7* expression on the salinity and peroxide tolerance of *E. coli* were determined. In a controlled trial, as shown in Fig. 9A, there was no apparent difference in cell survival between the MsHSP17.7-expressing strain and the E2-expressing strain except at 2 and 4 h. However, the MsHSP17.7-expressing strain showed increased (*P* < 0.05) survival after treatment with 200 mM NaCl compared to the vector control throughout the entire treatment period (Fig. 9B). Similarly, the MsHSP17.7-expressing strain showed tolerance to 15 mM H_2_O_2_ treatment (Fig. 9C).

### Analysis of transgenic *Arabidopsis* under stress conditions

**Fig. 10:**
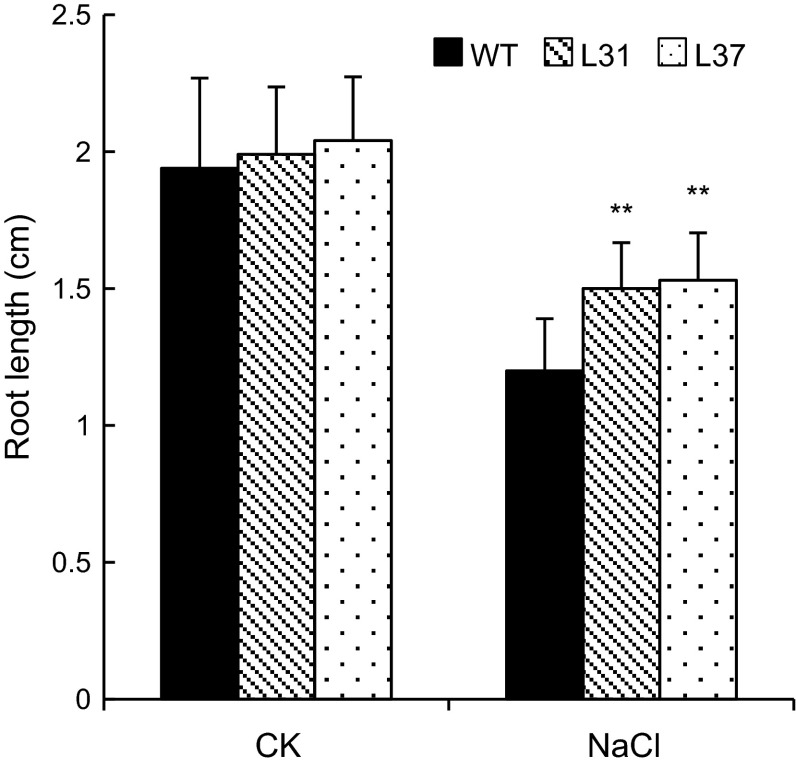
Root length of transgenic and wild-type *Arabidopsis* under salt stress (150 mM NaCl). *Double asterisk* indicates highly significant difference compared with wild-type *Arabidopsis* according to the LSD *t* test (*P* < 0.01). *Vertical bars* indicate the mean ± SE of three independent experiments

In the transgenic lines L31 and L37 under normal growth conditions, the root lengths were 1.99 and 2.04 cm, respectively, as shown in Fig. 10, which were not significantly different from the wild-type line. Under salt stress, the root lengths of transgenic *Arabidopsis* lines were 1.5 and 1.53 cm, which were 0.3 and 0.33 cm longer than the wild-type line (*P* < 0.01).

**Fig. 11:**
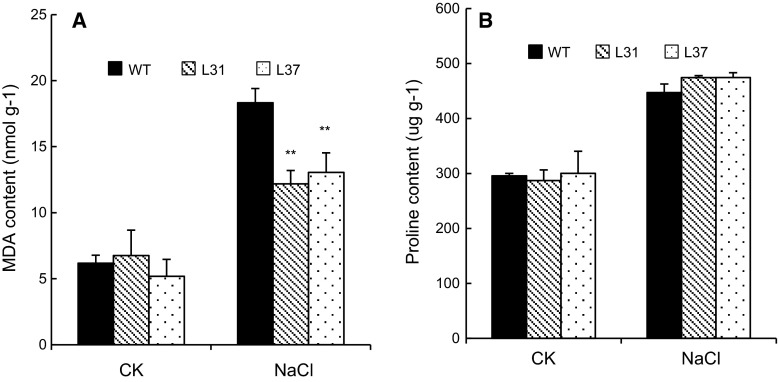
MDA (**A**) and proline (**B**) contents were measured in transgenic and wild-type *Arabidopsis* under salt stress condition (200 mM NaCl). *Double asterisk* indicates highly significant difference compared with wild-type *Arabidopsis* according to the LSD *t* test (*P* < 0.01). *Vertical bars* indicate the mean ± SE of three independent experiments

Under control conditions, the MsHSP17.7-overexpressing lines did not show significant differences in proline or MDA levels compared with wild-type *Arabidopsis* (Fig. 11). However, after treatment with 200 mM NaCl, the MDA content of the L31 and L37 lines were 12.2 and 13.0 nmol g^−1^, respectively, and were significantly lower that of the wild-type line (Fig. 11A). There was no significant difference in the proline level between the transgenic and wild-type lines (Fig. 11B).

## Discussion

Strategies employed by higher plants for surviving adverse conditions include behavioral adaptations, morphological changes and physiological regulation [18]. The role of sHSPs in protecting cells against damage related to abiotic and biotic stresses has been well demonstrated in organisms ranging from fungi [19, 20] to plants [16, 21]. The functions of sHSPs include binding to unfolded proteins and regulating their intracellular distribution, protein degradation and signal transduction, allowing cell survival under stress conditions [9]. In spite of the considerable research on the role of *sHSP* in response to environmental stresses in diverse plants, few studies on alfalfa *sHSP* have been reported. We cloned and characterized the *MsHSP17.7* gene from alfalfa. A phylogenetic tree analysis divided 50 sHSPs into six families, of which Classes CI, CII and CIII are localized in the cytosol or nucleus [4]. Additionally, subcellular localization studies demonstrated that *MsHSP17.7* is located in the cytosol. Combined with the evolutionary tree analysis, we propose that MsHSP17.7 is a cytosolic Class II sHSP.

*sHSP* genes are highly induced under conditions ranging from abiotic exposure to biotic stresses. It has been reported that *A. thaliana* CI *sHSPs* (*Hsp17.4CI*, *Hsp17.6ACI*, *Hsp17.6BCI*, and *Hsp17.6CCI*) are expressed during heat stress, osmotic stress, oxidative stress, UV-B exposure and other abiotic stressors [9]. *HSP*s are induced by heat, cold, drought, and oxidative and salt stresses in *Oryza sativa* [22]. In addition, Neta-Sharir demonstrated that tomato chloroplast *sHSP21* is induced by heat treatment in leaves [23].

In this paper, the abundantly increased *MsHSP17.7* mRNA indicated adaptation of the plant to the adverse environments. *sHSP* genes also respond to osmotic and salt stress. Ruibal et al. [24] reported that *PpHsp16.4* was up-regulated after exposure to various abiotic stress factors, including strong light, heat, salt and osmotic stress. In this study, *MsHSP17.7* mRNA was up-regulated by NaCl and PEG treatments, suggesting that *MsHSP17.7* might play a key role in osmotic and salt stress. *AtHSP15.7*, a peroxisomal *sHSP*, has been shown to be strongly induced by both oxidative and heat stress [11]. However, the analysis of an *Arabidopsis* cytosolic class II *sHSP*, *AtHSP17.6A*, revealed that this *sHSP* was induced by osmotic but not oxidative stress [25]. In our study, *MsHSP17.7* mRNA expression was regulated in alfalfa roots under oxidative stress, but its levels remained stable in the stems and leaves. The At-HSP17.6A protein was not detected in PEG-treated *Arabidopsis* plants, whereas *At*-*HSP17.6A* mRNA was induced [15]. In our study, *MsHSP17.7* was induced after chronic drought treatment. Therefore, *MsHSP17.7* was presumed to be involved in the drought response and to play a crucial role in plant stress tolerance. Under high temperature, drought, high salt concentration, or exposure to various pathogens [26], the primary function of this sHSP is to promote refolding of non-native proteins that have been denatured under stress condition.

Previous studies have demonstrated that the constitutive overexpression of *sHSPs* in plants is associated with enhanced resistance to abiotic stress [15, 16]. In this study, *MsHSP17.7*-expressing *E. coli* showed increased survival following salt stress and peroxide stress, respectively, compared with controls. In *Arabidopsis*, overexpression of wheat chloroplastic *sHSP26* results in improved heat tolerance [27]. In this study, the transgenic *Arabidopsis* seedlings exhibited significantly longer root, lower MDA content and similar proline content compared to wild-type *Arabidopsis* under high salinity stress. The lower MDA content indicated that less damage occurred in the plant. Some researchers argue that proline is a compatible osmolyte that accumulates in plant cells in response to salt stress [28], but others favor the view that proline is simply a stress-induced product [29]. In the present study, there was no marked difference in the proline content between transgenic and wild-type *Arabidopsis*.

In conclusion, we showed that *MsHSP17.7* is localized in the cytoplasm and is induced by heat shock, high salinity, peroxide and drought stress. Expression of *MsHSP17.7* in transgenic *E. coli* and *A. thaliana* indicated that it could enhance salt tolerance.

## Electronic supplementary material

Below is the link to the electronic supplementary material.

Supplementary material 1 (DOCX 407 kb)
