# Supplementary material for: Molecular cloning and characterization of the MsHSP17.7 gene from Medicago sativa L
Source: Mol Biol Rep. 2016 May 19;43:815–26. doi: 10.1007/s11033-016-4008-9 (PMC4947596; doi:10.1007/s11033-016-4008-9)
Supplement: Supplementary file 1 — Supplementary material 1 (DOCX 407 kb) [file 11033_2016_4008_MOESM1_ESM.docx]

**Supplementary photographs and captions**


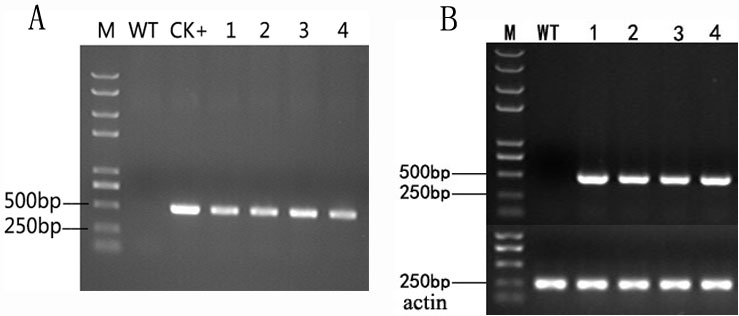

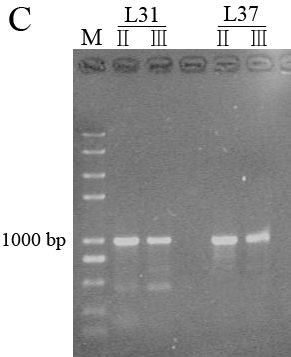


**Supplementary figure 1** Identification of transgenic *Arabidopsis* with *MsHSP17.7* by PCR (A), RT-PCR (B) and TAIL-PCR (C). *Lane M* DNA marker trans2000 plusII, *lane WT* wild-type *Arabidopsis*, *lane CK*+ pBI121-MsHSP17.7 vector, *lane 1-4* transgenic lines, actin represent an internal reference control. In figure C, each set of two lanes contains products from secondary (II) and tertiary (III) reactions. The BLAST results showed that *MsHSP17.7*was intergrated in chromosomes 1 (GenBank: CP002684.1; range, 258,118,99-258,124,42, 99%) of L31 and chromosomes 4 (GenBank: CP002687.1; range, 167,073,28-167,085,36, 99%) of L37.


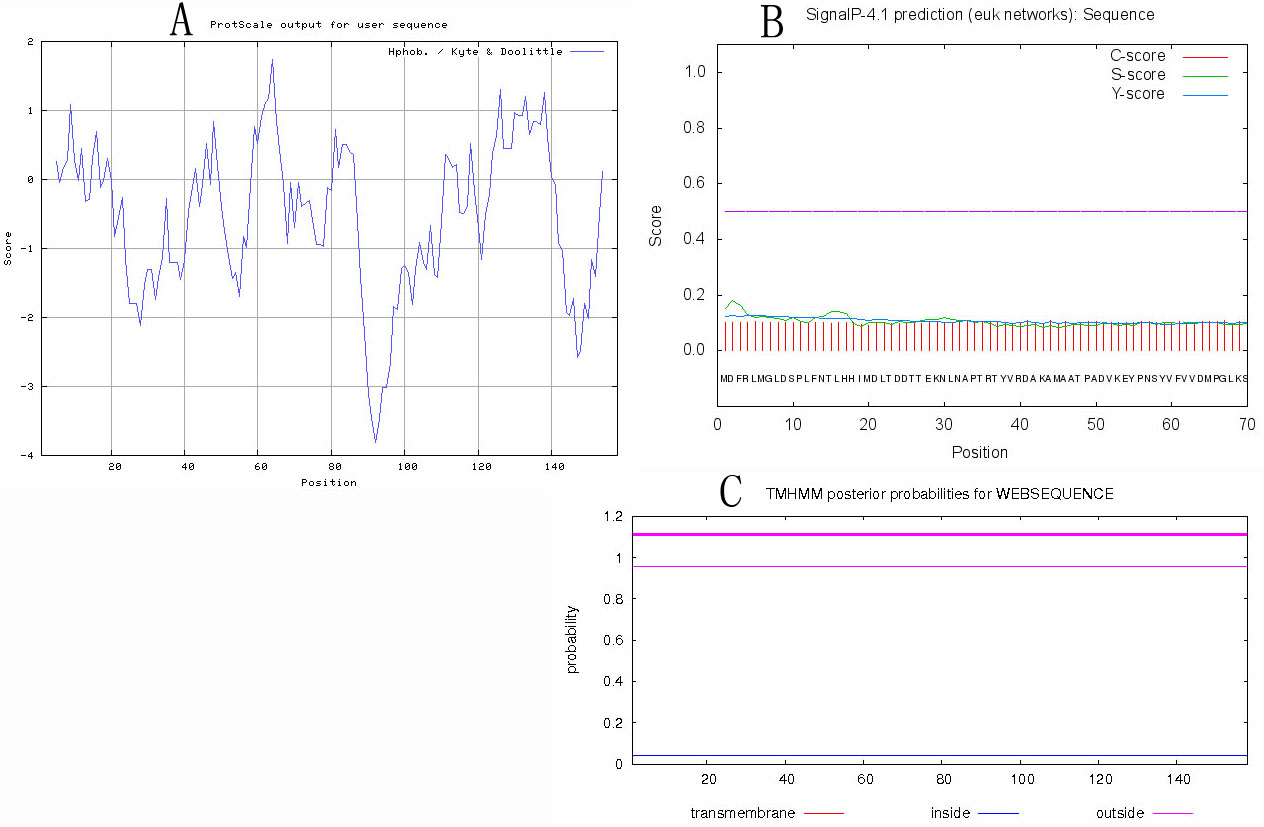


**Supplementary figure 2** The bioinformatic analysis of MsHSP17.7 hydrophobicity, signal prediction and transmembrane motif prediction. A represents the prediction of MsHSP17.7’s hydrophilicity, negative value indicates polar amino acids and positive value indicates nonpolar amino acids. ProtScale analysis indicated that MsHSP17.7 was a hydrophilic protein. B represents prediction of signal peptide. C-score (raw cleavage site score), S-score (signal peptide score) and Y-score (combined cleavage site score) determined there is no signal peptides in MsHSP17.7. C represents transmembrane structure prediction by TMHMM. The red line denotes the possibility of transmembrane amino acids; the blue line denotes the possibility of amino acid within membrane; the purple line denotes the possibility of amino acid outside membrane. Taken together, these analyses indicate that MsHSP17.7 is not a membrane-binding protein.


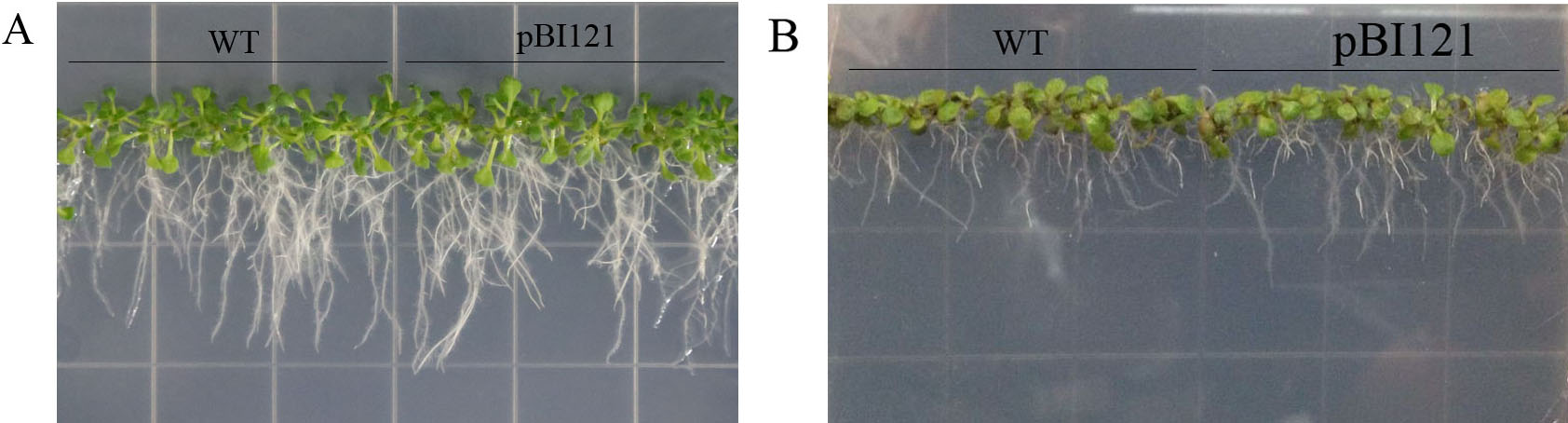


**Supplementary figure 3** 20-day seedlings of WT and transgenic *Arabidopsis* with empty vector under normal condition (**A**) and 100 mM NaCl treatment (**B**). were exhibited in the figure. WT indicates wild type Arabidopsis thaliana, pBI121 indicates the transgenic *Arabidopsis* with empty vector.


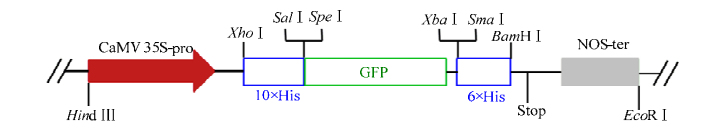


**Supplementary figure 4** The pA7-GFP, 4.9 kb, contains CaMV 35S-promoter, histidines and the green fluorescent protein (GFP) gene. The insertion of the Hind Ⅲ/EcoR Ⅰ fragment from vector pBI-35s-10H-GFP-JFH1 is inserted into pUC18.
